# German Multicenter Study Analyzing Antimicrobial Activity of Ceftazidime-Avibactam of Clinical Meropenem-Resistant *Pseudomonas aeruginosa* Isolates Using a Commercially Available Broth Microdilution Assay

**DOI:** 10.3390/antibiotics11050545

**Published:** 2022-04-19

**Authors:** Jana Manzke, Raphael Stauf, Bernd Neumann, Ernst Molitor, Gunnar Hischebeth, Michaela Simon, Jonathan Jantsch, Jürgen Rödel, Sören L. Becker, Alexander Halfmann, Thomas A. Wichelhaus, Michael Hogardt, Annerose Serr, Christina Hess, Andreas F. Wendel, Ekkehard Siegel, Holger Rohde, Stefan Zimmermann, Jörg Steinmann

**Affiliations:** 1 Institute of Clinical Hygiene, Medical Microbiology and Infectiology, Paracelsus Medical University, Klinikum Nürnberg, 90419 Nuremberg, Germany; jana.manzke@klinikum-nuernberg.de (J.M.); raphael.stauf@gmail.com (R.S.); bernd.neumann@klinikum-nuernberg.de (B.N.); 2 Institute of Medical Microbiology, Immunology and Parasitology, University Hospital Bonn, 53127 Bonn, Germany; molitor@uni-bonn.de (E.M.); hischebeth@microbiology-bonn.de (G.H.); 3 Institute of Clinical Microbiology and Hygiene, Regensburg University Hospital, 93053 Regensburg, Germany; michaela.simon@klinik.uni-regensburg.de (M.S.); jonathan.jantsch@klinik.uni-regensburg.de (J.J.); 4 Institute for Medical Microbiology, Immunology, and Hygiene, University Hospital Cologne and Faculty of Medicine, University of Cologne, 50937 Cologne, Germany; 5 Institute of Medical Microbiology, Jena University Hospital, 07743 Jena, Germany; juergen.roedel@med.uni-jena.de; 6 Institute of Medical Microbiology and Hygiene, Saarland University, 66421 Homburg, Germany; soeren.becker@uks.eu (S.L.B.); alexander.halfmann@uks.eu (A.H.); 7 German National Consiliary Laboratory on Cystic Fibrosis Bacteriology, Institute of Medical Microbiology and Infection Control, University Hospital Frankfurt, Goethe University, 60590 Frankfurt am Main, Germany; thomasa.wichelhaus@kgu.de (T.A.W.); michael.hogardt@kgu.de (M.H.); 8 Department for Medical Microbiology and Hygiene, University Hospital Freiburg, 79106 Freiburg, Germany; annerose.serr@uniklinik-freiburg.de (A.S.); christina.hess@uniklinik-freiburg.de (C.H.); 9 Institute of Hygiene, Cologne Merheim Medical Centre, University Hospital of Witten/Herdecke, 51058 Cologne, Germany; wendela@kliniken-koeln.de; 10 Institute for Medical Microbiology, University Medical Center, Johannes Gutenberg University Mainz, 55131 Mainz, Germany; ekkehard.siegel@unimedizin-mainz.de; 11 Institute for Medical Microbiology, Virology and Hygiene, University Medical Center Hamburg-Eppendorf, 20251 Hamburg, Germany; rohde@uke.de; 12 Department of Infectious Diseases, University Hospital Heidelberg, 69120 Heidelberg, Germany; stefan.zimmermann@med.uni-heidelberg.de; 13 Institute of Medical Microbiology, University Hospital Essen, 45122 Essen, Germany

**Keywords:** ceftazidime-avibactam, susceptibility testing, carbapenemases

## Abstract

Multidrug resistance is an emerging healthcare issue, especially concerning *Pseudomonas aeruginosa*. In this multicenter study, *P. aeruginosa* isolates with resistance against meropenem detected by routine methods were collected and tested for carbapenemase production and susceptibility against ceftazidime-avibactam. Meropenem-resistant isolates of *P. aeruginosa* from various clinical materials were collected at 11 tertiary care hospitals in Germany from 2017–2019. Minimum inhibitory concentrations (MICs) were determined via microdilution plates (MICRONAUT-S) of ceftazidime-avibactam and meropenem at each center. Detection of the presence of carbapenemases was performed by PCR or immunochromatography. For meropenem-resistant isolates (*n* = 448), the MIC range of ceftazidime-avibactam was 0.25–128 mg/L, MIC_90_ was 128 mg/L and MIC_50_ was 16 mg/L. According to EUCAST clinical breakpoints, 213 of all meropenem-resistant *P. aeruginosa* isolates were categorized as susceptible (47.5%) to ceftazidime-avibactam. Metallo-β-lactamases (MBL) could be detected in 122 isolates (27.3%). The MIC range of ceftazidime-avibactam in MBL-positive isolates was 4–128 mg/L, MIC_90_ was >128 mg/L and MIC_50_ was 32 mg/L. There was strong variation in the prevalence of MBL-positive isolates among centers. Our in vitro results support ceftazidime-avibactam as a treatment option against infections caused by meropenem-resistant, MBL-negative *P. aeruginosa*.

## 1. Introduction

Multidrug-resistant (MDR) Gram-negative infections are emerging worldwide. Multi-resistant and extensively drug-resistant *Pseudomonas aeruginosa* phenotypes are a major cause for nosocomial, difficult-to-treat infections [1]. The novel beta-lactam/beta-lactamase inhibitor combinations ceftolozane-tazobactam and ceftazidime-avibactam (CZA) show high in vitro activity against *P. aeruginosa*, including ceftazidime- and carbapenem-resistant isolates [2,3]. Avibactam, as a novel non-β-lactam β-lactamase inhibitor, targets the active site of serine β-lactamases, resulting in the inhibition of extended-spectrum β-lactamases, AmpC β-lactamases and class A/D carbapenemases (e.g., KPC and OXA-48) [4]. However, as metallo-β-lactamases (MBL) production is one of the most common mechanisms of carbapenem-resistance in *P. aeruginosa*, ceftolozane-tazobactam and CZA are not able to inhibit *P. aeruginosa* isolates carrying MBLs [5].

In a recent European multicenter study including 1673 MDR *P. aeruginosa* isolates, CZA showed antipseudomonal activity in 71.7% [6]. This finding highlights the need for routine antimicrobial susceptibility testing of the second-generation β-lactam/β-lactamase inhibitor combinations in MDR phenotypes [7]. Gradient or disk diffusion tests for CZA have been evaluated in comparison to the broth microdilution (BMD) method. While disk diffusion testing tends to overestimate resistance, the categorical agreement for the gradient tests was over 90% compared to BMD in most studies [8,9,10].

The study presented here was done in collaboration with the microbiology laboratories of 11 German tertiary care hospitals with the intention to investigate the following objectives: (a) implementation of a standardized platform for susceptibility testing based on the BMD method in routine diagnostics to determine the minimum inhibitory concentrations for ceftazidime-avibactam; (b) analyzing the CZA in-vitro activity against meropenem (MEM)-resistant *P. aeruginosa* isolates; (c) evaluation of the most prevalent carbapenemases, including MBLs, in MEM-resistant *P. aeruginosa* isolates in all participating centers and correlating the corresponding CZA MICs.

## 2. Results

In total, 448 MEM-resistant isolates, with varying numbers in the respective study centers (Table 1), were included in this study. Further, testing for MBL revealed different abundances in participating institutions.

The overall prevalence of carbapenemase genes among the included isolates was 28% (126/448), with four identifications of non-MBL carbapenemases (OXA-48, *n* = 2; KPC-2, *n* = 1; GES, *n* = 1). A wide variation in the proportion of MBL-positive isolates was observed among individual centers, ranging from 0 to 75% (Table 1). Some centers had low amounts of MBL-positive *P. aeruginosa* (Table 1; centers A, I and K), whereas in other centers high proportions were identified (Table 1, centers E and G). In 91% of the MBL-positive isolates, Verona integron-encoded metallo-β-lactamase (VIM) was detected. In the remaining 9%, an Imipenemase (IMP) or the New Delhi metallo-β-lactamase (NDM) were found. The origins of MBL-positive isolates were from respiratory specimens (23.8%), urine (19.7%), wound swabs (9.8%), blood culture (7.4%), rectal swab (3.2%) or not specified materials (36.1%).

**Figure 1:**
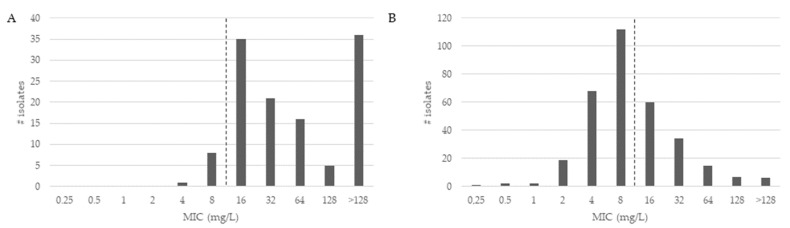
Distribution of antibiotic minimum inhibitory concentrations (MICs) for ceftazidime-avibactam of meropenem-resistant *Pseudomonas aeruginosa* isolates. For antimicrobial susceptibility testing purposes, the concentration of avibactam was fixed at 4 mg/L. The pointed line depicts the CZA-resistance breakpoint (susceptible ≤ 8). (**A**) shows the MIC distribution for MBL-positive isolates; (**B**) shows the MIC distribution for MBL-negative isolates.

The MICs for CZA are shown for MBL-positive and MBL-negative subgroups in Figure 1.

As shown in Figure 1, the CZA MICs of MBL-positive isolates (Figure 1A) were higher compared to MICs of MBL-negative isolates (Figure 1B). The CZA MIC_90_ was determined at 128 mg/L and the CZA MIC_50_ at 16 mg/L. Overall, 213 of all MEM-resistant *P. aeruginosa* isolates were categorized as susceptible (47.5%) to CZA. For the group of MBL-positive isolates, the CZA MIC_90_ was >128 mg/L and MIC_50_ was at 32 mg/L. Of all MBL-positive *P. aeruginosa* isolates, nine isolates carrying the VIM gene were categorized as susceptible (7.4%) to CZA. In the 326 MBL-negative isolates, the MIC_90_ of CZA was 32 mg/L and the MIC_50_ was at 8 mg/L. Further, 204 of all MBL-negative *P. aeruginosa* isolates were categorized as susceptible (62.6%) to CZA.

## 3. Discussion

A recent study investigated the antimicrobial resistance of 2588 *P. aeruginosa* isolates over a period of 20 years. A significant increase in multidrug-resistant over time was reported, indicating the need for novel therapeutic opportunities [11]. In this German multicentre study, 448 MEM-resistant *P. aeruginosa* isolates from clinical specimen were tested against CZA using the standardised susceptibility testing method MICRONAUT-S (MERLIN Diagnostika GmbH, Bornheim, Germany), based on broth microdilution plates. Another recent study from Germany investigated 72 ceftazidime-resistant *P. aeruginosa* isolates towards CZA, of which 33 were resistant against imipenem and meropenem [12]. In total, 54.8% of ceftazidime and carbapenem-resistant *P. aeruginosa* isolates were susceptible to CZA, which is comparable to our results. In a German monocentric study, 112 XDR *P. aeruginosa* isolates had a susceptibility rate of 49.1% to CZA [9].

We found an MBL in 27% of isolates, with up to 75% in one centre. Carbapenemase production in the isolates varied geographically on a national level but did not show a specific trend towards the north/south or the east/west direction. Similarly, a recent multi-national study (ERACE Global Surveillance program) analysed 807 carbapenem-resistant *P. aeruginosa* isolates. In total, 33% carbapenemase-positive isolates were found via phenotypic methods [13]. The Middle East and Africa showed the highest resistance rates, with 43% and 66% for CZA, respectively. In contrast, a study from the US from 2017 found only 4% of VIM in 290 meropenem-resistant *P. aeruginosa* isolates, indicating a high geographic heterogeneity of MBL-positive *P. aeruginosa* [14].

Easy-to-perform and reliable susceptibility testing of *P. aeruginosa* is important for diagnostic microbiological laboratories and the appropriate management of infectious diseases. Despite commercially available gradient and disk diffusion tests, semi-automated platforms are now able to provide CZA MICs. Studies have shown that gradient diffusion tests demonstrated good accuracy compared to BMD, whereas disk diffusion showed higher MICs [9,15]. Recently, the performance of the Vitek2 (bioMerieux, Nürtingen, Germany) system was analysed for reliability of CZA susceptibility testing [16]. A rate of 18.1% of misclassification of susceptible strains was reported, and it was concluded that for Vitek2 and MIC gradient tests a control with the broth microdilution (BMD) method is needed when MIC values are close to a breakpoint [15]. Here, we only used one commercially available method for susceptibility testing based on microdilution, which does not allow any interpretation of the reliability of MICs in comparison to the gold standard BMD method according to EUCAST. However, in comparison with the results from other German surveillance studies based on the BMD method, our data are comparable [11,14]. Further, a recent study presented comparable good performance and trustworthy results for accurate MIC determination of CZA of another commercially available method (MicroScan WalkAway, Beckman Coulter, Brea, CA, USA) [17].

To the best of our knowledge, this is the first German multicentre study investigating CZA MICs with a high number of meropenem-resistant isolates. However, the present study has several limitations. Whole-genome-sequencing (WGS) to investigate clonal relatedness for excluding multi-copy strains with the same genetic background was not part of the study. Furthermore, exploring the detailed resistance mechanisms of the isolates (e.g., efflux pumps) by WGS to investigate specific in-vitro activities of CZA would have improved the work. Further, the data only reflects in-vitro testing and does not reflect in-vivo susceptibility or treatment recommendations. However, the presented multicentre study depicts a sound image of carbapenem-resistant MDR *P. aeruginosa* in several tertiary care hospitals and further outlines possibilities for a routine surveillance test strategy for Germany.

In conclusion, CZA exhibits a high level of activity against meropenem-resistant, metallo-β-lactamase-negative *P. aeruginosa* in Germany. The prevalence of MBLs differed in the contributing centres, highlighting the need for accurate antimicrobial susceptibility testing.

## 4. Materials and Methods

### 4.1. Bacterial Isolates

All clinical samples were subjected to a conventional microbiological diagnosis before use. The study did not use demographic data about patients, nor did it result in any additional constraints for the patients. Because of the retrospective nature of the study, all data were anonymously analysed without the need for patient consent. All procedures and methods were carried out in accordance with approved guidelines.

In total, 640 non-duplicate clinical isolates of *P. aeruginosa* with phenotypic resistance against meropenem as inclusion criterion were collected between 2017 and 2019 from 11 German tertiary care hospitals. Species identification was performed at all centers by matrix-assisted laser desorption ionization time-of-flight (MALDI-TOF) mass spectrometry (Bruker Daltonik GmbH, Bremen, Germany, or bioMerieux, Nürtingen, Germany).

### 4.2. PCR- and Immunochromatography Screening for Resistance Genes

Detection of carbapenemases was done by multiplex PCR or immunochromatography-based tests according to the manufacturer’s recommendations at each center.

Center A: in-house PCR including NDM, KPC, VIM, IMP, OXA-23, OXA-48, OXA-58, OXA-72; center B: in-house PCR including VIM, OXA-48, NDM, KPC, GES; center C: PCR Xpert Carba-R including NDM, IMP-1, OXA-48, VIM, KPC (v2, Cepheid GmbH, Krefeld, Germany) and immunochromatography RESIST-4 O.K.N.V. including VIM, NDM, KPC, OXA-48-like (Coris BioConcept, Gembloux, Belgium); center D: NG-Test CARBA 5 including KPC, OXA-48-like, VIM, IMP, NDM (NG Biotech, Guipry, France) and in-house PCR including VIM, NDM, OXA-48, KPC, IMP; center E: NG-Test CARBA 5 including KPC, OXA-48-like, VIM, IMP, NDM (NG Biotech, Guipry, France); center F: real-time PCR Xpert Carba-R including NDM, OXA-48, VIM, KPC (v2, Cepheid, Sunnyvale, CA, USA); center G: eazyplex Superbug basic including KPC, NDM, VIM, OXA-48-like, OXA-181 (Amplex Diagnostics GmbH, Gießen, Germany); center H: AID Carbapenemase PCR Kit including AIM, BIC, DIM, GIM, IMI, IMP, KPC, NDM-1, NMC-A, OXA-48, SIM, SPM, VIM (AID Autoimmun Diagnostika GmbH, Strassberg, Germany); center I: in-house assay including IMP, VIM, NDM, OXA-48-like, GES, NMC-A/IMI, BIC, SME; center J: Allplex-DR including IMP, KPC, NDM, OXA-48, VIM (Seegene Germany GmbH, Düsseldorf, Germany); center K: eazyplex Superbug CRE including KPC, NDM, VIM, OXA-48, OXA-181 CTX-M1, CTX-M9 (Amplex Diagnostics GmbH, Gießen, Germany).

### 4.3. Antimicrobial Susceptibility Testing

The MICs were determined by the BMD method using the MICRONAUT-S system (MERLIN Diagnostika GmbH, Bornheim, Germany) containing two-fold dilutions of CZA (0.125/4–128/4 mg/L) and of MEM (0.0625–128 mg/L) in each center, according to the manufacturer´s recommendations. After 18–24 h, incubation plates were read digitally with a photometer. The *P. aeruginosa* ATCC strain 27853 was used as quality control strain. CLSI and EUCAST clinical breakpoints (v. 12.0, 2022) were applied for interpretation of MICs (MEM: S ≤ 2; R > 8 mg/L; CZA: R > 8 mg/L).

In the final analysis, only confirmed MEM-resistant *P. aeruginosa* isolates were included. The numbers of isolates provided by each participating center are shown in Table 1.

## Figures and Tables

**Table 1:** Proportion of metallo-β-lactamase (MBL)-positive isolates to total number of meropenem-resistant isolates by center.

Center	Total Isolates	MBL-Positive (%)	VIM-Positive	IMP-Positive	NDM-Positive
A	63	4 (6.3)	3	0	1
B	62	24 (38.7)	18	4	2
C	31	4 (12.9)	4	0	0
D	14	3 (21.4)	3	0	0
E	44	33 (75.0)	33	0	0
F	43	11 (26.6)	11	0	0
G	36	23 (63.9)	19	4	0
H	28	8 (28.6)	8	0	0
I	16	1 (6.3)	1	0	0
J	62	11 (17.7)	11	0	0
K	49	0 (0.0)	0	0	0
∑ 11	∑ 448	∑ 122	∑ 111	∑ 8	∑ 3

Abbreviations: MBL: metallo-β-lactamase; VIM: Verona integron-encoded metallo-β-lactamase; IMP: Imipenemase; NDM: New Delhi metallo-β-lactamase.

## Data Availability

Not applicable.
